# Atrioventricular Nodal Reentrant Tachycardia in Very Elderly Patients: A Single-center Experience

**DOI:** 10.19102/icrm.2020.110202

**Published:** 2020-02-15

**Authors:** Khalil Kanjwal, Shaffi Kanjwal, Mohammed Ruzieh

**Affiliations:** ^1^McLaren Greater Lansing Hospital, Central Michigan University, Lansing, MI, USA; ^2^St. Mary’s of Michigan, Central Michigan University, Lansing, MI, USA; ^3^Penn State Heart and Vascular Institute, Penn State Milton S. Hershey Medical Center, Hershey, PA, USA

**Keywords:** Ablation, atrioventricular nodal reentrant tachycardia, elderly, slow-pathway conduction

## Abstract

We present a series of elderly patients older than 80 years who had recurrent palpitations for decades and who were subsequently diagnosed with atrioventricular (AV) nodal reentrant tachycardia (AVNRT). Through a retrospective chart analysis, we identified 12 patients (nine females and three males) aged 88 years ± 3.7 years (range: 80–92 years) seen at our center from 2015 to 2016 for recurrent palpitations and supraventricular tachycardia (SVT) who were ultimately diagnosed with AVNRT. These patients had palpitations and had been treated for anxiety and panic attacks for decades. They underwent electrophysiology (EP) study and successful ablation of the slow pathway. The demographic data, symptoms, and EP characteristics during the EP studies of the patients were evaluated. All 12 patients experienced palpitations and all but three had documented SVT on a loop recorder or an event monitor. During EP study, all patients displayed slow-pathway conduction. Nine patients demonstrated discontinuous AV nodal conduction curves, while three showed continuous AV nodal conduction curves. The observed tachycardia rates were 496.7 ms ± 25.7 ms. Three patients had atrial fibrillation (AF), which was noted during monitoring with the implanted loop recorders. Tachycardia was induced with both burst atrial pacing and atrial extrastimuli in five patients and with extrastimuli only in two patients. In five patients, no tachycardia induction was noted, but these individuals showed evidence of dual AV node physiology. Successful elimination of residual slow-pathway conduction postablation and/or noninducibility of tachycardia in the postablation period were achieved in all patients. All patients remained symptom-free over a period of one year. The patients who had AF in addition to AVNRT also did not present any recurrent AF following AVNRT ablation but are being monitored for recurrence. AVNRT in elderly people is often confused with panic attacks; hence, reports of panic attacks in elderly people should be properly evaluated for an arrhythmic etiology.

## Introduction

Atrioventricular (AV) node reentrant tachycardia (AVNRT) is the most common supraventricular tachycardia (SVT). It usually affects children and young adults.^1–4^ We present a series of elderly patients who had recurrent palpitations but who had been incorrectly labeled as having panic attacks for decades. They subsequently were diagnosed with AVNRT and underwent ablation of the slow pathway. In this report, we present the clinical and electrophysiology (EP) characteristics of these patients.

## Methods

In a retrospective chart analysis, we identified 12 patients aged 88 years ± 3.7 years who were diagnosed with AVNRT. These patients had palpitations and had been treated for anxiety and panic attacks for decades. They underwent long-term cardiac monitoring for better symptom–rhythm correlation, EP study, and successful ablation of the slow pathway. We collected data pertaining to the patients’ demographics, symptoms, and EP characteristics during the EP studies as well as their symptoms after ablation.

### Long-term event monitoring

A 30-day event monitor (MCOT™ Monitor; CardioNET, Malvern, PA, USA) was deployed in patients who continued to have palpitations. In case the event monitor did not capture any symptomatic episodes, implantable loop recorders (Reveal LINQ™; Medtronic, Minneapolis, MN, USA) were also inserted.

### Electrophysiology study

Patients were selected for EP study if they had a documented episode of SVT and/or were experiencing recurrent palpitations. All patients were brought to the EP laboratory in a postabsorptive fasting state. Conscious sedation was initiated and maintained throughout the procedure. The patients were then prepared and draped in the usual sterile fashion. The right femoral site was locally anesthetized and four venous sheaths (one 6-French, two 5-French, and one SR-0; St. Jude Medical, St. Paul, MN, USA) were placed using a modified Saldinger technique with a 5-French micropuncture kit under ultrasound guidance. EP catheters were advanced under fluoroscopic guidance to the high right atrium, His bundle, right ventricle apex, and coronary sinus (CS).

### Electrophysiology study protocol

Baseline intervals were measured. Atrial burst pacing was performed up to the AV block cycle length (AVBCL) or to induction of the tachycardia. Atrial extrastimuli were performed to look for AV jump, the AV node effective refractory period (AVNERP), tachycardia induction, or the atrial effective refractory period. If patients were not inducible for any tachycardia, isoproterenol up to a maximum dose of 10 mg was initiated and the induction protocol was repeated.

### Diagnosis of atrioventricular nodal reentrant tachycardia

**Figure 1:**
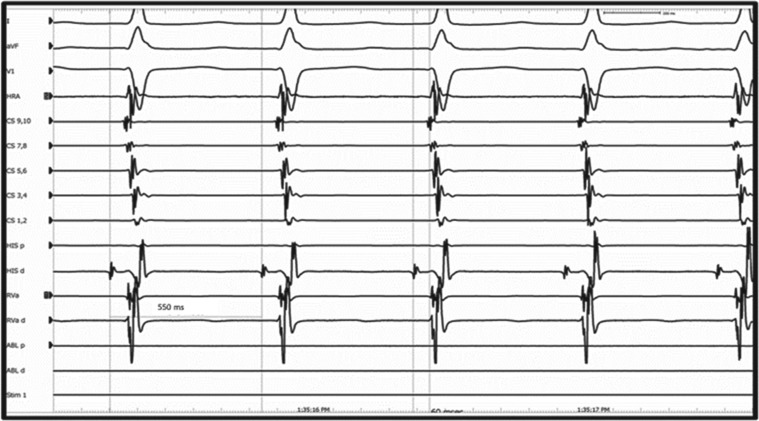
A short VA interval (< 70 ms) induced during EP study in a patient with typical AVNRT (slow–fast).

Patients were diagnosed with AVNRT as a mechanism of their tachycardia if they had evidence of dual AV node physiology or an inducible AVNRT during the EP study. Dual AV node physiology was diagnosed if the patient demonstrated a 50-ms increase in the A–H interval with a 10-ms decrement in atrial extrastimuli.^4,5^ Separately, if an SVT was induced, AVNRT was diagnosed using standard criteria. During tachycardia, the septal ventriculoatrial (VA) interval was less than 70 ms. Ventricular entrainment during tachycardia demonstrated a V–A–H–V response and a long postpacing interval–tachycardia cycle length (; 115 ms). If the tachycardia was terminated during tachycardia, the transition zone criterion was used.^3–6^ Some of the characteristics of typical AVNRT are shown in **Figures 1 through 3**.

### Ablation strategy

Slow-pathway ablation was performed by localizing it anterior to the CS ostium. Radiofrequency (RF) ablation was conducted using a 4-mm nonirrigated-tip catheter (Biosense Webster, Diamond Bar, CA, USA). RF ablation was completed in a temperature-control mode up to a maximum of 45 W. Intermittent junctional beats were seen during ablation. If intermittent junctional beats were not observed, the catheter was moved superiorly to the midseptal level. If junctional beats were still not observed, programmed stimulation was repeated to assess for residual slow-pathway conduction or reinduction of the tachycardia. Programmed stimulation was performed both on and off isoproterenol. Patients were followed up with in the arrhythmia clinic at six and 12 months for any recurrence of symptoms.^7^

## Results

Twelve patients (nine females and three males) were included in the analysis; their clinical characteristics are summarized in **Table 1**.

### Symptoms

All included patients had been experiencing palpitations for decades at the time of this study. These palpitations initially lasted for a few minutes to hours. Most of these patients had previously been to the emergency room (ER) and, by the time they arrived in the ER, the palpitations had already subsided, with all of them found to have sinus tachycardia while at the ER. All 12 patients had a history of more than five ER visits and, each time, they were found to be in sinus tachycardia. They were subsequently misdiagnosed as having panic attacks and were given antianxiety medications. They were ultimately seen in our arrhythmia clinic and underwent long-term cardiac monitoring either using a 30-day event monitor or with insertion of a loop recorder.

### Cardiac monitoring

Three patients were found during the study period to have a documented episode of a regular, narrow complex tachycardia. In nine patients, the 30-day event monitor could not capture a symptomatic episode of palpitations, and they subsequently underwent loop recorder insertion. Documented episodes of SVT captured on said loop recorders between two and nine months occurred in all nine patients. Three of these patients were also found to have atrial fibrillation (AF). These patients were started on oral anticoagulation.

### Electrophysiology study results

Findings obtained during EP testing are summarized in **Table 1**. All patients demonstrated slow-pathway conduction. Nine patients had discontinuous AV node conduction curves with a clear jump and echo phenomenon, whereas three demonstrated continuous AV node conduction curves without A–H jump during decremental atrial pacing.

Tachycardia was induced with both burst atrial pacing and atrial extrastimuli in five patients and with extrastimuli only in two patients. In five patients, no induction of tachycardia was noted, although these individuals did show evidence of dual AV node physiology. Of the seven patients who were inducible for tachycardia, three required isoproterenol for tachycardia induction.

All 12 patients underwent slow-pathway ablation, which was localized in the posteroseptal location just superior to the CS ostium in eight patients. The slow pathway was localized in the midseptal location in four patients. Five patients had no intermittent junction noted during the ablation of the slow pathway. All patients presented successful elimination of slow-pathway conduction postablation and/or noninducibility of tachycardia in the postablation period both on and off isoproterenol using burst pacing and atrial extrastimuli. There were no complications observed immediately. Three patients had bruising noted at the site of catheter insertion one day after the procedure, but all cases resolved completely within a reasonable time frame.

### Follow-up

All patients were followed in the arrhythmia clinic for a period of one year. They remained symptom-free and had no recurrence of any arrhythmia. Nine patients with insertable loop recorders had no evidence of any arrhythmia at follow-up. Three patients who had AF as noted on loop recorders had no recurrence of their AF following ablation of the slow pathway. All patients were taken off their antianxiety medications and have done well. We continued oral anticoagulation in the three patients with AF.

## Discussion

SVT is a common arrhythmia and usually affects young people, with AVNRT being the most common SVT affecting young patients.^1–4^ The diagnosis of such is straightforward in most patients. We report on a series of 12 patients who had a long-standing history of palpitations and were wrongly labeled as having panic attacks; AVNRT was subsequently found to be the reason for the palpitations.

### Symptoms in our study population

The diagnosis of SVT and AVNRT, which is usually straightforward, can be elusive because episodes of the tachycardia may subside before the patient seeks medical help for the episode. Unfortunately, our study population had been experiencing palpitations for decades before a diagnosis was made. This could have been partly due to the nature of the arrhythmia in question, which is short-lived and may terminate before the patients were seen in the ER. A common theme in our study population was the abrupt onset of symptoms and termination before arriving at the ER.

The known female predominance in AVNRT persisted in older age as well. Almost all patients were found to have sinus tachycardia when they arrived in the ER in years past. This led to the incorrect attribution of their symptoms to either anxiety or panic attacks. Although SVT and especially AVNRT are usually seen in young adults, physicians need to be aware that elderly patients can experience such arrhythmias as well. The reason for sinus tachycardia could be a component of anxiety secondary due to palpitations and rapid heart rates from AVNRT even if the episode terminated. This led to incorrect labeling of these patients as having panic attacks. ER physicians need to be aware of these clinical scenarios.

### Electrophysiology characteristics

There were certain interesting findings noted during EP study in our cohort. We found AVNRT with a heart rate as slow as 110 bpm. This could be due to the existence of a slower conduction system in this aged population, as was suggested by the longer AVBCL and AVNERP. Furthermore, tachycardia was not induced in five of 12 patients; however, they all demonstrated dual AV node physiology during EP study. Most of the patients required isoproterenol infusion for induction of the tachycardia, which was likely due to a slower baseline heart rate and long AVBCL. Five patients had no intermittent junctional beats noted during slow-pathway ablation, but a repeat programmed stimulation after each set of ablations failed to demonstrate any residual slow-pathway conduction or an inducible tachycardia in these patients. Previous reports have demonstrated that intermittent junctional beats, although a marker of slow-pathway ablation, are not essential for successful AVNRT ablation.^7^ It is very important to repeat programmed stimulation after each series of ablations to demonstrate continued slow-pathway conduction or inducible tachycardia before attempting further ablation, especially in elderly patients with a prolonged A–H interval. This strategy is important to minimize the risk of complete heart block or a fast-pathway injury in such individuals.

Three patients had AF as noted using a loop recorder during the monitoring period. Elimination of slow-pathway conduction in these patients resulted in the resolution of their symptoms, and none of the three patients with AF showed such during follow-up to one year. In these individuals, we continued monitoring as well as anticoagulation given the risk of stroke. Patients with SVT (AVNRT or AVRT) have been shown to degenerate into AF and, interestingly, the elimination of an accessory pathway or slow-pathway conduction results in the resolution of AF in these individuals. In one study, a significant proportion of candidates who underwent AF ablation were inducible for SVT. SVT ablation showed a preventive effect on AF recurrence. The authors of this previous study suggested that these patients should be selected to undergo simpler ablation procedures tailored only toward the suppression of the triggering arrhythmia.^8^

### Role of long-term cardiac monitoring

Our patients had symptoms of palpitation for decades. Unfortunately, they were wrongly labeled as having anxiety and panic attacks. Prolonged monitoring with an event monitor or a loop recorder allowed for proper diagnosis and treatment in each patient. Patients with anxiety or panic attacks should be evaluated with a prolonged cardiac monitor if their symptoms do not respond to antianxiety medications. Due to the infrequent and short-lived nature of episodes of AVNRT, the diagnosis can often be challenging to make and possible only with prolonged monitoring with an implantable loop recorder. Successful ablation resulted in the complete elimination of symptoms of tachycardia and palpitations in our study group.

Of note, there is often a tendency to withhold invasive therapy in the elderly, for fear of complications. RF catheter ablation for supraventricular and ventricular arrhythmias has been shown to be not only effective but safe as well.^9,10^ In our study group, no major complications were noted. All patients had complete resolution of their palpitations. They were completely taken off their antianxiety medications as well.

Elderly patients should be evaluated for an arrhythmic etiology when reporting palpitations, as many may benefit from EP study and ablation. These procedures are safe and should be offered to all patients as one available management option regardless of patient age.

### Limitations

There are certain important limitations in this study design and analysis that should be highlighted. This was a nonrandomized, descriptive analysis involving a small group of highly selected patients. However, we believe that there are certain important points that were brought up in this study. All of our patients had a common story of long-standing palpitations that would terminate before arriving at the ER. All of these patients were found to have only sinus tachycardia and were thus wrongly labeled as having anxiety or panic attacks. Further, they were treated for these false panic attacks for decades; it was only after prolonged cardiac monitoring that they received a proper diagnosis. Therefore, the authors feel that this study supports the validity of the need to better evaluate patients who present with a similar clinical scenario.

## Conclusion

AVNRT in the elderly is often confused with panic attacks. Panic attacks in the elderly should be properly evaluated for an arrhythmic etiology with long-term cardiac monitoring either using an event monitor or a loop recorder, as these patients may benefit from subsequent RF ablation.

## Figures and Tables

**Figure 2:**
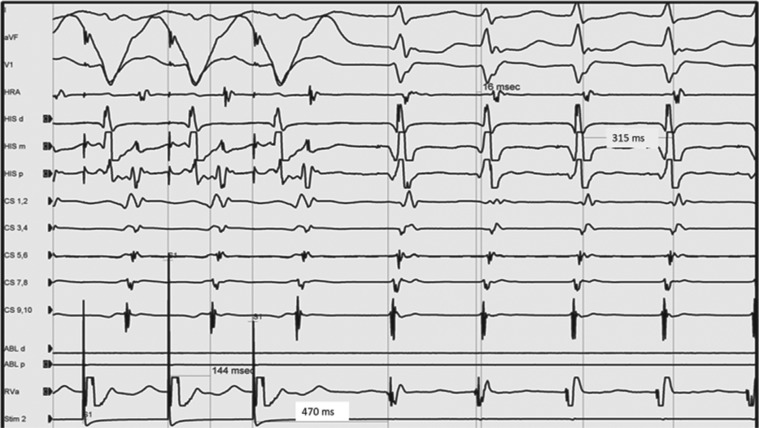
A postpacing interval–tachycardia cycle length of more than 115 ms during ventricular entrainment of SVT consistent with a typical AVNRT. All these features are consistent with a diagnosis of typical (slow–fast) AVNRT.

**Figure 3:**
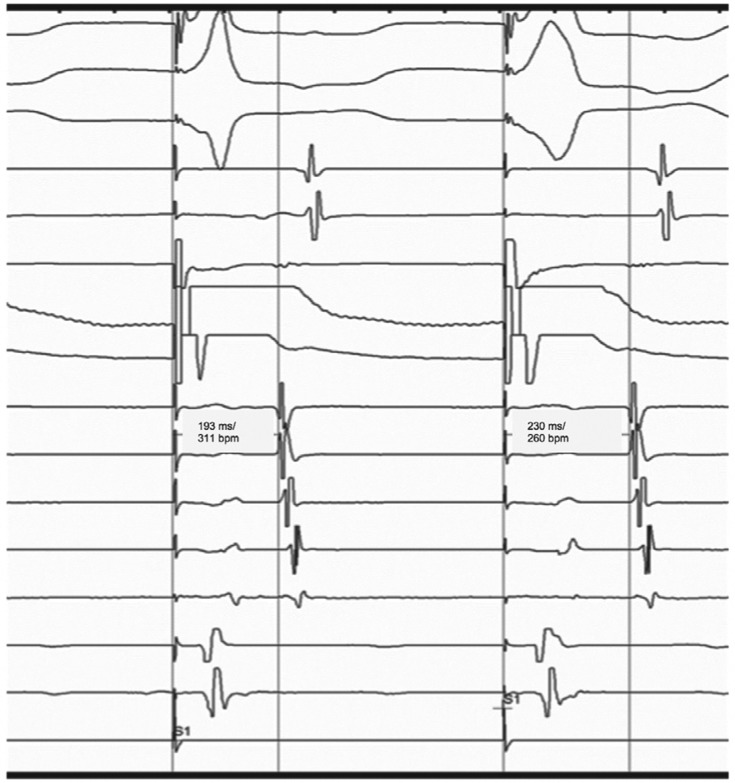
Nodal response seen on para-Hisian pacing suggestive of retrograde conduction through the AV node. Note the stimulation to an atrial electrogram time of 193 ms with His capture (narrow QRS) and 230 ms with a loss of His capture (wide QRS).

**Table 1:** Clinical and Electrophysiology Characteristics of the Study Population (n = 12)

Age, mean ± SD	85.0 ± 3.4 years
Female gender, n (%)	9 (75.0%)
PR interval, mean ± SD	215.4 ± 25.0 ms
QRS duration, mean ± SD	116.8 ± 15.9 ms
QT interval, mean ± SD	440.5 ± 31.4 ms
A–H interval, mean ± SD	100.3 ± 30.6 ms
H–V interval, mean ± SD	56.9 ± 8.0 ms
AVBCL interval, mean ± SD	388.3 ± 36.9 ms
VABCL interval, mean ± SD	457.5 ± 71.0 ms
ERP interval at 600-ms drive train, mean ± SD	419. 2 ± 25.0 ms
TCL interval, mean ± SD	496.7 ± 25.7 ms

AVBCL, atrioventricular block cycle length; ERP: effective refractory period; SD: standard deviation; VABCL: ventriculoatrial block cycle length.
